# Test-Retest Reliability of Velocity and Power in the Deadlift and Squat Exercises Assessed by the GymAware PowerTool System

**DOI:** 10.3389/fphys.2020.561682

**Published:** 2020-09-09

**Authors:** Jozo Grgic, Bela Scapec, Zeljko Pedisic, Pavle Mikulic

**Affiliations:** ^1^Institute for Health and Sport (IHES), Victoria University, Melbourne, VIC, Australia; ^2^Faculty of Kinesiology, University of Zagreb, Zagreb, Croatia

**Keywords:** velocity-based training, linear position transducer, movement velocity, resistance training, strength

## Abstract

We explored the test-retest reliability of velocity and power assessed by the GymAware PowerTool system (GYM) in the deadlift and squat by simulating a context with and without a familiarization session. Sixteen resistance-trained individuals completed three testing sessions. In all sessions, velocity and power were assessed by the GYM system in the deadlift and squat exercises with loads of 30, 45, 60, 75, and 90% of one-repetition maximum. The consistency of test results between the first session and the second session was considered to represent the reliability with no familiarization session. The consistency of test results between the second session and the third session was considered to represent the reliability with one familiarization session because the first session simulates a familiarization session. Intraclass correlation coefficients (ICCs) ranged 0.63–0.99 in the deadlift, and 0.78–0.99 in the squat. ICCs were higher than 0.75 for 93 and 100% of all deadlift and squat tests, respectively. For velocity and power, standard error of measurement ranged 0.03–0.08 m/s and 20–176 W, respectively. The coefficient of variation ranged 2.2–10.6% for the deadlift and 2.6–6.9% for the squat tests. Except for peak and mean velocity at 30% of 1RM in the squat, we found no significant improvements in reliability with a familiarization session. The test-retest reliability of velocity and power assessed by the GYM system was moderate-to-excellent for the deadlift and good-to-excellent for the squat. Reliability of velocity and power did not seem to improve with a familiarization session.

## Introduction

In recent years, velocity-based training (VBT) has gained substantial popularity (Pareja-Blanco et al., 2017; Sánchez-Moreno et al., 2020). VBT involves the performance of resistance exercises with maximal intended concentric velocity (Pareja-Blanco et al., 2017). In VBT, the training load is regulated based on velocity data (Pareja-Blanco et al., 2017; Sánchez-Moreno et al., 2020). Velocity in resistance exercise has been shown to accurately quantify the level of effort and fatigue during an exercise session and to estimate the proximity to muscle failure (Morán-Navarro et al., 2019). Linear position transducers (LPTs) are often used for measuring velocity in resistance exercise (Ruf et al., 2018; Courel-Ibáñez et al., 2019). LPTs are systems that measure velocity through a vertical displacement of a cable that is attached to the barbell (Courel-Ibáñez et al., 2019). One such system is GymAware PowerTool (GYM; Kinetic Performance Technologies, Canberra, Australia). This system consists of a unit with a cable that measures velocity and transfers the data via Bluetooth to a tablet/computer. Using the GYM app, the user can instantaneously obtain velocity data.

For both training and testing purposes, it is important to know the reliability of velocity and power assessed by the GYM system. Test-retest reliability denotes the consistency of test results across repeated measurements and shows how much test results are affected by measurement error (Atkinson and Nevill, 1998). Depending on the training phase and exercise selection, resistance training aimed at improving muscular power is recommended to be performed using loads ranging from 30–90% of one-repetition maximum (1RM) (Haff and Nimphius, 2012). Therefore, when exploring the reliability of velocity and power in resistance exercise, it is important to use a broad spectrum of loads. Two studies explored the reliability of velocity and power assessed by the GYM system while using different loads (Chéry and Ruf, 2019; Orange et al., 2020). In the studies, the participants performed repetitions at maximum intended concentric velocity, using loads in the 20–100% of 1RM range in the bench press, squat, and deadlift. The reliability of the GYM system for assessing velocity and power was deemed to be good at some loads [intraclass correlation coefficient (ICC) ≥ 0.75]. However, at very low loads (20 and 40% of 1RM) and maximal loads (100% of 1RM), the reliability of the GYM system for assessing velocity and power was reported to be less reliable, as correlation ranged 0.42–0.71 and coefficient of variation (CV) for some outcomes was as high as 17% (Chéry and Ruf, 2019; Orange et al., 2020). In both studies, the participants were initially familiarized with performing the concentric phase of the selected exercises with maximal intentional velocity. After that, the reliability of velocity and power was assessed by the GYM system in two testing sessions (test and retest). In these studies, the initial familiarization session was not included in the reliability analyses. This should be considered when interpreting the findings, as familiarization with the exercise protocol may impact reliability of the test (Currell and Jeukendrup, 2008). Indeed, current recommendations are to familiarize the participants with the exercise protocol at least once before the main testing session (Currell and Jeukendrup, 2008).

We aimed to explore the reliability of velocity and power assessed by the GYM system in the deadlift and squat exercises with loads in the 30–90% of 1RM range by simulating a context with and without a familiarization session. We hypothesized that the reliability of velocity and power quantified using GYM would be greater at higher loads and that it would improve with test familiarization.

## Methods

### Experimental Design

All participants attended four sessions, including a preliminary testing session and three main testing sessions. In the preliminary testing session, the participants were tested for their 1RM in the deadlift and squat exercises. In this preliminary session, the GYM system was not used, and we did not provide any suggestions regarding velocity to the participants. In the first, second, and third main testing sessions, velocity and power in these two exercises were measured with loads corresponding to 30, 45, 60, 75, and 90% of 1RM. These loads have been chosen, because training for muscular power is commonly recommended to be performed at loads in the 30–90% of 1RM range (Haff and Nimphius, 2012). In the three sessions, the GYM system was used to measure velocity and power and the participants were instructed to lift the loads at maximum intended velocity. All sessions took place between 11 a.m. and 6 p.m., and always at the same time of day (±1 h) for each participant, to avoid the possible confounding effect of circadian variation (Grgic et al., 2019). The testing sessions were performed 4–7 days apart. For the day before each testing session, the participants were advised not to perform any strenuous exercise, and not to substantially change their usual food intake. Participants verbally confirmed compliance with these recommendations before starting each testing session.

### Participants

The study sample included men and women who were free of musculoskeletal injuries and who were resistance-trained (defined as possessing the ability to lift at least 100% of their body mass in the squat and deadlift). All participants also had a minimum of 1 year of resistance training experience, with a minimum weekly training frequency of two times per week (on most weeks). Ten men and six women volunteered to take part in the study, and they completed all testing sessions (mean ± SD of age: 26 ± 5 years). The *a priori* sample size calculation was based on the formula number 3 from Bonett (2002), which indicated that 16 participants are needed if the expected ICC is 0.95 and the desired precision of its 95% confidence interval (CI) is ± 0.10. The expected ICC of 0.95 was based on findings of a previous study on reliability of mean velocity at 60% of 1RM in the deadlift (Chéry and Ruf, 2019). The Committee for Scientific Research and Ethics of the Faculty of Kinesiology at the University of Zagreb provided ethical approval for the study (approval number: 74/2020), and all participants provided their written informed consent before enrolling in the study.

### Deadlift and Squat Performance

For the deadlift, the participants performed the “conventional” variation of the exercise. The participants were required to grip the barbell using either a fully pronated grip or a mixed grip. Grip variation was based on personal preference, but all participants were required to keep the grip type consistent across all sessions. Feet were required to be placed approximately hip and shoulder-width apart. In the starting position, hips were required to be lower than the shoulder but higher than the knees, with the chest elevated. Participants were required to extend at the knees and hips to raise the barbell off the floor up to an upright standing position. In the back squat exercise, the participants were required to place the barbell in the “high-bar” position. After lifting the load from the squat rack, the participants were required to descend downward (were their lowest position included reaching at least the parallel between the thigh and the floor) and return to the upright standing position. An experienced rater monitored the squat depth for consistency.

### 1RM Test

In 1RM testing, the deadlift exercise was performed first, followed by the squat. Before starting with the 1RM testing protocol, the participants were provided with 10 min of self-selected warm-up. After the warm-up, the 1RM testing protocol started. The participants first performed three sets leading up to 1RM attempts. These sets were performed for 8–10 repetitions, 3–5 repetitions, and one repetition, with 50, 75, and 95% of the participant’s expected 1RM, respectively. Then, the participants performed 1RM attempts with increases in load in each subsequent attempt. Testing was performed until the participants were not able to complete the 1RM attempt. All 1RM values were determined within five attempts. Three-minute and 10 min rest periods were provided between sets and exercises, respectively. 1RM values were used to determine the loads calculated as percentages of 1RM for the subsequent sessions. In this session, the GYM system was not used, and no suggestions were provided to the participants regarding velocity of movement.

### Test-Retest Reliability

In the main testing sessions, velocity and power in the deadlift and squat was assessed with loads corresponding to 30, 45, 60, 75, and 90% of 1RM. These exercise sessions were identical in structure, and the same rater conducted all assessments. After arriving at the laboratory, the participants first performed 10 min of self-selected warm-up. The participants were instructed to keep the warm-up consistent across all sessions. After the warm-up was completed and before the testing sessions began, the participants were instructed to perform each repetition across all loading schemes with maximal intended concentric velocity while maintaining eccentric control. The participants first performed the deadlift followed by the squat exercise. In both exercises, the load was first set to 30% of 1RM and progressively increased to 90% of 1RM. At all loads up to 75% of 1RM, the participants completed one set of three consecutive repetitions, whereas at 90% of 1RM, the participants completed one set with a single repetition. The participants rested for 3 min between sets and for 10 min between exercises.

### GYM System

The floor unit of the GYM system has a spring-powered retractable cable with a tether that was attached to the barbell perpendicular to the floor (Orange et al., 2020). With the movement of the barbell the GYM software automatically detects the start of the concentric phase. The GYM software down-samples the collected data to 50 points per second (Dorrell et al., 2019). Data recorded with the floor unit were transmitted via Bluetooth to a tablet (iPad; Apple, Inc., Cupertino, CA, United States). We focused on four main outcomes: (1) mean velocity (m/s); (2) peak velocity (m/s); (3) mean power (W); and (4) peak power (W). Data on all outcomes were collected during the concentric phase of the repetition. A description of calculations used by the GYM system for velocity and power data is available elsewhere (Dorrell et al., 2019).

### Statistical Analysis

All comparisons were conducted between the first and second, and the second and third testing sessions. The consistency of test results between the first session and the second session represents the reliability in the context with no familiarization session. The consistency of test results between the second session and the third session was considered to represent the reliability in the context with one familiarization session, because the first session simulates a familiarization session. The presence of systematic changes in test results across measurements was explored using one-way repeated measures analysis of variance (ANOVA). Relative effect sizes were calculated using Cohen’s *d* for repeated measures and interpreted as trivial (<0.20), small (0.20–0.49), medium (0.50–0.79), and large (≥0.80), according to Cohen (1992). Mean differences between the testing sessions were also calculated, together with their 95% CIs. These procedures have been performed in IBM SPSS software, version 23 (SPSS Inc., an IBM Company, Chicago, IL, United States). Reliability was explored using ICC from the two-way mixed model for single measure and representing absolute agreement, that is, type (A,1) case 3A, according to McGraw and Wong (1996). ICCs were interpreted as: “poor” (< 0.50), “moderate” (0.50–0.75), “good” (0.75–0.90), and “excellent” (>0.90) (Koo and Li, 2016). Within-participant variation in test results across measurements was determined by calculating CV. We also calculated the standard error of measurement (SEM). To examine possible effects of a familiarization session on reliability, we tested whether ICCs for the first vs. second testing session significantly differed from ICCs for the second vs. third testing session. This was done by calculating the differences between the pairs of ICCs, together with their non-parametric bootstrap 95% CIs obtained from ordinary resampling using the adjusted bootstrap percentile (BCa) method. These analyses were performed using the “psych” (Revelle, 2019) and “boot” (Canty and Ripley, 2020) packages in R version 3.4 (The R Foundation for Statistical Computing, Vienna, Austria). The statistical significance threshold was set at *p* < 0.05.

## Results

Mean ± SD for all performance outcomes recorded at all loads are reported in Table 1 (deadlift) and Table 2 (squat). For the deadlift, there were no significant differences between the first and second testing sessions in any of the analyzed outcomes (*p* > 0.05 for all). At 30, 45, 60, 75, and 90% of 1RM, ICCs ranged 0.81–0.96, 0.82–0.99, 0.83–0.99, 0.63–0.99, and 0.66–0.93, respectively (Table 3). CVs ranged 2.2–10.6%. For mean velocity, SEMs ranged 0.03–0.05 m/s, while for peak velocity, they ranged 0.03–0.08 m/s. For mean power, SEMs ranged 20–56 W, while for peak power, they ranged 44–115 W.

**TABLE 1:** Differences between testing sessions in velocity and power of the deadlift exercise.

Variable and load	Testing session (mean ± standard deviation)			*p*-value		Mean difference (95% CI)		Cohen’s *d*	
	First session (1)	Second session (2)	Third session (3)	1 vs. 2	2 vs. 3	1 vs. 2	2 vs. 3	1 vs. 2	2 vs. 3
Peak velocity 30% 1RM (m/s)	1.67 ± 0.23	1.69 ± 0.19	1.67 ± 0.23	0.472	0.382	0.02 (−0.04, 0.08)	−0.02 (−0.08, 0.03)	0.10	−0.11
Mean velocity 30% 1RM (m/s)	0.98 ± 0.11	0.99 ± 0.10	0.98 ± 0.12	0.515	0.289	0.01 (−0.02, 0.04)	−0.02 (−0.05, 0.01)	0.10	−0.16
Peak power 30% 1RM (W)	929 ± 379	956 ± 383	921 ± 361	0.520	0.333	27 (−53, 107)	−35 (−105, 34)	0.07	−0.10
Mean power 30% 1RM (W)	419 ± 152	428 ± 152	417 ± 151	0.455	0.286	8 (−13, 30)	−11 (−30, 8)	0.06	−0.07
Peak velocity 45% 1RM (m/s)	1.47 ± 0.18	1.47 ± 0.17	1.46 ± 0.19	0.943	0.507	0.00 (−0.05, 0.05)	−0.01 (−0.05, 0.02)	0.01	−0.07
Mean velocity 45% 1RM (m/s)	0.86 ± 0.09	0.86 ± 0.09	0.85 ± 0.10	0.862	0.597	0.00 (−0.03, 0.03)	−0.01 (−0.03, 0.02)	0.03	−0.08
Peak power 45% 1RM (W)	1153 ± 451	1156 ± 444	1115 ± 412	0.921	0.286	4 (−68, 75)	−41 (−114, 32)	0.01	−0.10
Mean power 45% 1RM (W)	543 ± 185	544 ± 183	541 ± 190	0.874	0.719	1 (−13, 15)	−3 (−18, 12)	0.01	−0.02
Peak velocity 60% 1RM (m/s)	1.26 ± 0.15	1.24 ± 0.14	1.23 ± 0.13	0.127	0.482	−0.02 (−0.04, 0.00)	−0.01 (−0.03, 0.01)	−0.13	−0.06
Mean velocity 60% 1RM (m/s)	0.73 ± 0.07	0.73 ± 0.09	0.73 ± 0.07	0.498	0.592	0.01 (−0.01, 0.03)	−0.01 (−0.03, 0.02)	0.10	−0.09
Peak power 60% 1RM (W)	1248 ± 452	1194 ± 403	1176 ± 387	0.078	0.346	−54 (−110, 2)	−18 (−54, 18)	−0.13	−0.05
Mean power 60% 1RM (W)	614 ± 201	620 ± 211	614 ± 201	0.430	0.533	6 (−9, 21)	−6 (−23, 12)	0.03	−0.03
Peak velocity 75% 1RM (m/s)	0.99 ± 0.14	0.98 ± 0.13	1.00 ± 0.11	0.425	0.173	−0.01 (−0.04, 0.02)	0.02 (−0.01, 0.05)	−0.09	0.17
Mean velocity 75% 1RM (m/s)	0.59 ± 0.07	0.57 ± 0.10	0.60 ± 0.06	0.566	0.134	−0.01 (−0.05, 0.03)	0.03 (−0.01, 0.06)	−0.13	0.31
Peak power 75% 1RM (W)	1131 ± 377	1113 ± 379	1135 ± 372	0.272	0.275	−18 (−48, 13)	22 (−16, 61)	−0.05	0.06
Mean power 75% 1RM (W)	615 ± 210	621 ± 215	628 ± 206	0.571	0.534	6 (−14, 26)	7 (−15, 30)	0.03	0.04
Peak velocity 90% 1RM (m/s)	0.76 ± 0.18	0.74 ± 0.14	0.77 ± 0.16	0.507	0.321	−0.02 (−0.07, 0.03)	0.02 (−0.02, 0.07)	−0.11	0.16
Mean velocity 90% 1RM (m/s)	0.43 ± 0.09	0.42 ± 0.08	0.44 ± 0.08	0.606	0.263	−0.01 (−0.04, 0.03)	0.02 (−0.01, 0.04)	−0.11	0.20
Peak power 90% 1RM (W)	1002 ± 436	989 ± 347	1033 ± 404	0.747	0.233	−13 (−91, 65)	44 (−26, 114)	−0.03	0.12
Mean power 90% 1RM (W)	536 ± 210	538 ± 198	553 ± 194	0.933	0.349	2 (−37, 41)	15 (−16, 46)	0.01	0.08

*1RM, one-repetition maximum; p-value, probability of type I error from repeated measures univariate analysis of variance (ANOVA); CI, confidence interval.*

**TABLE 2:** Differences between testing sessions in velocity and power of the squat exercise.

Variable and load	Testing session (mean ± standard deviation)			*p*-value		Mean difference (95% CI)		Cohen’s *d*	
	First session (1)	Second session (2)	Third session (3)	1 vs. 2	2 vs. 3	1 vs. 2	2 vs. 3	1 vs. 2	2 vs. 3
Peak velocity 30% 1RM (m/s)	1.63 ± 0.19	1.64 ± 0.20	1.60 ± 0.19	0.610	0.014	0.02 (−0.04, 0.07)	−0.04 (−0.07, −0.01)	0.08	−0.22
Mean velocity 30% 1RM (m/s)	1.00 ± 0.13	0.99 ± 0.13	0.97 ± 0.13	0.882	0.018	0.00 (−0.04, 0.04)	−0.03 (−0.04, −0.01)	−0.02	−0.20
Peak power 30% 1RM (W)	2299 ± 770	2353 ± 790	2235 ± 750	0.278	0.057	55 (−40, 150)	−118 (−231, 0)	0.07	−0.15
Mean power 30% 1RM (W)	1137 ± 360	1137 ± 360	1100 ± 348	0.993	0.008	0 (−38, 37)	−37 (−60, −13)	0.00	−0.10
Peak velocity 45% 1RM (m/s)	1.49 ± 0.18	1.49 ± 0.17	1.48 ± 0.15	0.814	0.648	0.00 (−0.03, 0.04)	−0.01 (−0.04, 0.03)	0.03	−0.05
Mean velocity 45% 1RM (m/s)	0.88 ± 0.12	0.87 ± 0.10	0.86 ± 0.10	0.406	0.797	−0.01 (−0.04, 0.02)	0.00 (−0.03, 0.02)	−0.11	−0.03
Peak power 45% 1RM (W)	2433 ± 813	2436 ± 814	2412 ± 750	0.929	0.529	−3 (−62, 68)	−24 (−97, 49)	0.00	−0.03
Mean power 45% 1RM (W)	1157 ± 384	1148 ± 376	1133 ± 353	0.501	0.351	−9 (−36, 17)	−15 (−45, 15)	−0.02	−0.04
Peak velocity 60% 1RM (m/s)	1.35 ± 0.13	1.33 ± 0.18	1.34 ± 0.16	0.362	0.672	−0.02 (−0.06, 0.02)	0.01 (−0.03, 0.05)	−0.12	0.06
Mean velocity 60% 1RM (m/s)	0.75 ± 0.10	0.74 ± 0.10	0.74 ± 0.10	0.541	0.767	−0.01 (−0.03, 0.01)	0.00 (−0.02, 0.02)	−0.06	−0.03
Peak power 60% 1RM (W)	2451 ± 791	2400 ± 826	2414 ± 794	0.241	0.781	−51 (−132, 31)	14 (−83, 111)	−0.06	0.02
Mean power 60% 1RM (W)	1104 ± 360	1098 ± 368	1094 ± 360	0.730	0.806	−6 (−38, 26)	−4 (−37, 28)	−0.02	−0.01
Peak velocity 75% 1RM (m/s)	1.23 ± 0.13	1.19 ± 0.16	1.21 ± 0.15	0.017	0.246	−0.05 (−0.08, −0.01)	0.02 (−0.01, 0.05)	−0.32	0.13
Mean velocity 75% 1RM (m/s)	0.62 ± 0.08	0.61 ± 0.08	0.61 ± 0.10	0.618	1.000	−0.01 (−0.02, 0.01)	0.00 (−0.02, 0.02)	−0.06	0.00
Peak power 75% 1RM (W)	2469 ± 798	2334 ± 814	2398 ± 790	0.003	0.174	−135 (−208, −62)	64 (−24, 153)	−0.17	0.08
Mean power 75% 1RM (W)	1013 ± 333	996 ± 327	1007 ± 345	0.326	0.611	−17 (−51, 16)	11 (−30, 52)	−0.05	0.03
Peak velocity 90% 1RM (m/s)	1.15 ± 0.18	1.14 ± 0.14	1.11 ± 0.16	0.593	0.181	−0.01 (−0.06, 0.03)	−0.03 (−0.07, 0.01)	−0.08	−0.20
Mean velocity 90% 1RM (m/s)	0.51 ± 0.08	0.53 ± 0.08	0.51 ± 0.10	0.285	0.059	0.02 (−0.01, 0.04)	−0.02 (−0.04, 0.00)	0.18	−0.25
Peak power 90% 1RM (W)	2500 ± 906	2431 ± 830	2350 ± 797	0.285	0.182	−69 (−190, 53)	−82 (−196, 33)	−0.08	−0.10
Mean power 90% 1RM (W)	931 ± 320	957 ± 322	918 ± 317	0.363	0.085	26 (−28, 80)	−38 (−79, 2)	0.08	−0.12

*1RM: one-repetition maximum; p-value: probability of type I error from repeated measures univariate analysis of variance (ANOVA); CI: confidence interval.*

**TABLE 3:** Reliability of repetition velocity in the deadlift.

Variable and load	First vs. second testing session			Second vs. third testing session			Diff (95% CI)
	ICC (95% CI)	CV	SEM	ICC (95% CI)	CV	SEM	
Peak velocity 30% 1RM	0.84 (0.66, 0.93)	3.60%	0.08 (m/s)	0.88 (0.72, 0.95)	3.60%	0.07 (m/s)	0.03 (−0.12, 0.18)
Mean velocity 30% 1RM	0.81 (0.60, 0.92)	3.60%	0.05 (m/s)	0.84 (0.65, 0.93)	3.90%	0.04 (m/s)	0.03 (−0.17, 0.20)
Peak power 30% 1RM	0.91 (0.80, 0.96)	7.00%	115 (W)	0.93 (0.83, 0.97)	6.10%	100 (W)	0.02 (−0.01, 0.06)
Mean power 30% 1RM	0.96 (0.90, 0.98)	4.60%	31 (W)	0.97 (0.92, 0.99)	4.10%	28 (W)	0.01 (−0.01, 0.04)
Peak velocity 45% 1RM	0.84 (0.66, 0.93)	4.10%	0.07 (m/s)	0.92 (0.82, 0.97)	2.80%	0.05 (m/s)	0.08 (−0.04, 0.22)
Mean velocity 45% 1RM	0.82 (0.61, 0.92)	3.90%	0.04 (m/s)	0.84 (0.66, 0.93)	3.30%	0.04 (m/s)	0.03 (−0.31, 0.19)
Peak power 45% 1RM	0.95 (0.89, 0.98)	6.50%	103 (W)	0.94 (0.86, 0.97)	5.50%	105 (W)	−0.01 (−0.11, 0.04)
Mean power 45% 1RM	0.99 (0.97, 1.00)	3.90%	20 (W)	0.99 (0.97, 0.99)	3.30%	22 (W)	0.00 (−0.02, 0.01)
Peak velocity 60% 1RM	0.95 (0.88, 0.98)	2.20%	0.03 (m/s)	0.95 (0.88, 0.98)	2.20%	0.03 (m/s)	0.00 (−0.08, 0.06)
Mean velocity 60% 1RM	0.83 (0.64, 0.93)	3.60%	0.03 (m/s)	0.81 (0.61, 0.92)	3.50%	0.04 (m/s)	−0.02 (−0.32, 0.12)
Peak power 60% 1RM	0.96 (0.92, 0.98)	4.10%	81 (W)	0.98 (0.96, 0.99)	3.00%	52 (W)	0.02 (−0.01, 0.11)
Mean power 60% 1RM	0.99 (0.97, 1.00)	3.20%	22 (W)	0.99 (0.96, 0.99)	2.80%	26 (W)	0.00 (−0.03, 0.01)
Peak velocity 75% 1RM	0.91 (0.80, 0.96)	3.20%	0.04 (m/s)	0.88 (0.74, 0.95)	3.80%	0.04 (m/s)	−0.03 (−0.08, 0.14)
Mean velocity 75% 1RM	0.63 (0.29, 0.83)	6.40%	0.05 (m/s)	0.71 (0.42, 0.87)	6.10%	0.04 (m/s)	0.08 (−0.10, 0.38)
Peak power 75% 1RM	0.99 (0.97, 0.99)	3.90%	44 (W)	0.98 (0.95, 0.99)	4.60%	56 (W)	−0.01 (−0.02, 0.01)
Mean power 75% 1RM	0.98 (0.96, 0.99)	4.30%	30 (W)	0.98 (0.94, 0.99)	4.30%	33 (W)	0.00 (−0.03, 0.01)
Peak velocity 90% 1RM	0.79 (0.57, 0.91)	8.30%	0.08 (m/s)	0.80 (0.58, 0.91)	7.20%	0.07 (m/s)	0.01 (−0.19, 0.16)
Mean velocity 90% 1RM	0.66 (0.35, 0.84)	10.60%	0.05 (m/s)	0.77 (0.52, 0.90)	7.70%	0.04 (m/s)	0.10 (−0.19, 0.33)
Peak power 90% 1RM	0.92 (0.83, 0.97)	8.80%	112 (W)	0.93 (0.83, 0.97)	8.10%	101 (W)	0.00 (−0.09, 0.06)
Mean power 90% 1RM	0.93 (0.84, 0.97)	9.30%	56 (W)	0.95 (0.88, 0.98)	7.70%	44 (W)	0.02 (−0.01, 0.05)

*1RM, one-repetition maximum; CI, confidence interval; ICC, intraclass correlation coefficient; CV, coefficient of variation; SEM, standard error of measurement; Diff, difference between ICC (second vs. third testing) and ICC (first vs. second testing).*

For the deadlift, there were no significant differences between the second and third testing sessions in any of the analyzed outcomes (*p* > 0.05 for all). At 30, 45, 60, 75, and 90% of 1RM, ICCs ranged 0.84–0.97, 0.84–0.99, 0.81–0.99, 0.71–0.98, and 0.77–0.95, respectively (Table 3). We found no significant differences between ICCs in the first vs. second testing session and ICCs in the second vs. third testing session. CVs ranged 2.2–8.1%. For mean velocity, SEM was 0.04 m/s for all loads, while for peak velocity, SEMs ranged 0.03–0.07 m/s. For mean power, SEMs ranged 22–44 W, while for peak power, they ranged 52–105 W.

For the squat, we found significant differences between the first and second testing sessions in peak velocity and peak power at 75% of 1RM (*p* = 0.017 and *p* = 0.003, respectively). For both outcomes, the values were higher in the first session. Mean ± SD of the difference between the sessions was 0.04 ± 0.07 m/s for peak velocity and 135 ± 150 W for peak power, respectively. No significant differences in any of the other analyzed outcomes were found (*p* > 0.05 for all comparisons). At 30, 45, 60, 75, and 90% of 1RM, ICCs ranged 0.80–0.98, 0.87–0.99, 0.86–0.98, 0.89–0.98, and 0.78–0.96, respectively (Table 4). CVs ranged 2.6–6.9%. For mean velocity, SEMs ranged 0.03–0.06 m/s, while for peak velocity, they ranged 0.05–0.08 m/s. For mean power, SEMs ranged 38–78 W, while for peak power, they ranged 94–176 W.

**TABLE 4:** Reliability of repetition velocity in the squat.

Variable and load	First vs. second testing session			Second vs. third testing session			Diff (95% CI)
	ICC (95% CI)	CV	SEM	ICC (95% CI)	CV	SEM	
Peak velocity 30% 1RM	0.82 (0.61, 0.92)	3.90%	0.08 (m/s)	0.95 (0.88, 0.98)	2.30%	0.04 (m/s)	0.13 (0.02, 0.39)
Mean velocity 30% 1RM	0.80 (0.59, 0.91)	4.70%	0.06 (m/s)	0.95 (0.89, 0.98)	2.40%	0.03 (m/s)	0.15 (0.04, 0.36)
Peak power 30% 1RM	0.97 (0.93, 0.99)	5.00%	137 (W)	0.96 (0.90, 0.98)	4.90%	162 (W)	−0.01 (−0.09, 0.02)
Mean power 30% 1RM	0.98 (0.95, 0.99)	4.80%	54 (W)	0.99 (0.98, 1.00)	2.80%	34 (W)	0.01 (0.00, 0.03)
Peak velocity 45% 1RM	0.92 (0.81, 0.96)	2.60%	0.05 (m/s)	0.91 (0.80, 0.96)	2.50%	0.05 (m/s)	−0.01 (−0.11, 0.07)
Mean velocity 45% 1RM	0.87 (0.72, 0.95)	3.30%	0.04 (m/s)	0.89 (0.76, 0.95)	3.30%	0.03 (m/s)	0.02 (−0.10, 0.18)
Peak power 45% 1RM	0.99 (0.97, 0.99)	3.60%	94 (W)	0.98 (0.96, 0.99)	3.50%	105 (W)	0.00 (−0.02, 0.01)
Mean power 45% 1RM	0.99 (0.98, 1.00)	3.00%	38 (W)	0.99 (0.97, 0.99)	3.10%	44 (W)	0.00 (−0.04, 0.00)
Peak velocity 60% 1RM	0.86 (0.70, 0.94)	4.10%	0.06 (m/s)	0.87 (0.72, 0.94)	3.50%	0.06 (m/s)	0.01 (−0.08, 0.08)
Mean velocity 60% 1RM	0.92 (0.82, 0.97)	3.10%	0.03 (m/s)	0.92 (0.82, 0.97)	3.40%	0.03 (m/s)	0.00 (−0.08, 0.11)
Peak power 60% 1RM	0.98 (0.95, 0.99)	4.50%	118 (W)	0.97 (0.93, 0.99)	4.80%	140 (W)	−0.01 (−0.02, 0.00)
Mean power 60% 1RM	0.98 (0.96, 0.99)	3.40%	46 (W)	0.98 (0.96, 0.99)	3.50%	47 (W)	0.00 (−0.01, 0.02)
Peak velocity 75% 1RM	0.89 (0.75, 0.95)	4.10%	0.05 (m/s)	0.91 (0.79, 0.96)	3.40%	0.05 (m/s)	0.02 (−0.06, 0.13)
Mean velocity 75% 1RM	0.89 (0.76, 0.95)	3.80%	0.03 (m/s)	0.87 (0.72, 0.94)	4.50%	0.03 (m/s)	−0.02 (−0.14, 0.12)
Peak power 75% 1RM	0.98 (0.96, 0.99)	5.40%	106 (W)	0.97 (0.94, 0.99)	5.20%	128 (W)	−0.01 (−0.03, 0.02)
Mean power 75% 1RM	0.98 (0.95, 0.99)	4.30%	48 (W)	0.97 (0.93, 0.99)	4.90%	59 (W)	−0.01 (−0.04, 0.01)
Peak velocity 90% 1RM	0.83 (0.64, 0.93)	5.10%	0.07 (m/s)	0.83 (0.64, 0.93)	4.80%	0.06 (m/s)	0.00 (−0.21, 0.07)
Mean velocity 90% 1RM	0.78 (0.54, 0.90)	6.70%	0.04 (m/s)	0.88 (0.74, 0.95)	5.90%	0.03 (m/s)	0.10 (−0.01, 0.28)
Peak power 90% 1RM	0.96 (0.90, 0.98)	6.10%	176 (W)	0.96 (0.90, 0.98)	5.60%	165 (W)	0.00 (−0.03, 0.03)
Mean power 90% 1RM	0.94 (0.86, 0.98)	6.90%	78 (W)	0.97 (0.92, 0.99)	5.70%	59 (W)	0.02 (−0.01, 0.07)

*1RM, one-repetition maximum; CI, confidence interval; ICC, intraclass correlation coefficient; CV, coefficient of variation; SEM, standard error of measurement; Diff, difference between ICC (second vs. third testing) and ICC (first vs. second testing).*

For the squat, we found significant differences between the second and third testing sessions in peak velocity, mean velocity, and mean power at 30% of 1RM (*p* = 0.014, *p* = 0.018 and *p* = 0.008, respectively). For all outcomes, the values were higher in the second session. Mean ± SD of the difference between the sessions was 0.04 ± 0.06 m/s, 0.02 ± 0.04 m/s and 37 ± 48 W for peak velocity, mean velocity, and mean power, respectively. No significant differences in any of the other analyzed outcomes were found (*p* > 0.05). At 30, 45, 60, 75, and 90% of 1RM, ICCs ranged 0.95–0.99, 0.89–0.99, 0.87–0.98, 0.87–0.97, and 0.83–0.97, respectively (Table 4). For peak and mean velocity at 30% of 1RM, ICCs for second vs. third testing session were significantly higher than ICCs for first vs. second testing session (Table 4). We found no significant differences between ICCs for other outcomes. CVs ranged 2.3–5.9%. For mean velocity, SEM was 0.03 m/s for all loads, while for peak velocity, SEMs ranged 0.04–0.06 m/s. For mean power, SEMs ranged 34–59 W, while for peak power they ranged 105–165 W.

## Discussion

The main finding of this study is that the test-retest reliability of velocity and power assessed by the GYM system could be considered moderate-to-excellent for the deadlift and good-to-excellent for the squat. Most ICCs were above 0.90, and CVs were consistently low (<5% for 57 out of 80 analyzed outcomes).

For the squat exercise, we found a significant difference between the first and second testing session in peak velocity and peak power at 75% of 1RM, with somewhat higher values in the first testing session. Significant differences were also found when comparing the second and third sessions, with higher values for peak velocity, mean velocity, and mean power at 30% of 1RM recorded in the second session. These results seem counterintuitive, as one might expect to observe a better performance in subsequent testing, as observed for weight lifted in the 1RM test (Grgic et al., 2020). Banyard et al. (2017) demonstrated that there are changes in the force-velocity relationship and its ability to predict 1RM in the back squat exercise across a week of training, even though the actual 1RM stayed relatively stable. Therefore, the differences in outcomes might partly stem from the incurred fatigue, especially if we consider that higher values (i.e., better performance) for all outcomes were observed in the first or second session. Despite the statistical significance of the differences, they might not be of substantial practical interest. Specifically, Cohen’s *d* for the four outcomes ranged 0.10–0.32 and these effects are classified as either “trivial” or “small”. Furthermore, for the deadlift, we did not observe significant differences across the testing sessions in any of the analyzed outcomes. While some results obtained with the GYM system for velocity in the back squat exhibited small systematic changes across repeated measurements, the practical importance of these findings remains to be determined.

ICCs ranged 0.78–0.99 and 0.63–0.99, in the squat and deadlift, respectively. ICCs were higher than 0.75 for 93% of the deadlift outcomes and 100% of the squat outcomes. They were higher than 0.90 for 60% of the deadlift outcomes and 68% of the squat outcomes. Based on these results, it can be concluded that the GYM system has moderate/good to excellent reliability for assessing velocity and power in the squat and deadlift. The reliability for velocity and power in the squat seems to be consistent across low, moderate, and high loads. The sample estimates of reliability for mean velocity at 75 and 90% of 1RM in the deadlift were somewhat lower than the sample estimates of reliability for other outcomes in this exercise. It might be that this test is somewhat less reliable when using higher loads in the deadlift, but this remains to be confirmed in future studies. For peak and mean velocity at 30% of 1RM, ICCs were significantly higher in the second vs. third testing session, suggesting that familiarization to the test has a positive impact on reliability. However, given that the improvement in reliability with a familiarization session was observed only for these two outcomes, this should be explored further in studies with larger samples.

Atkinson and Nevill (1998) suggested that extrapolating ICC values obtained in one study to a new sample of individuals involved in a given experiment should be performed with caution. These authors have also suggested that CV and SEM are reliability statistics that may be of great relevance for sports practitioners and researchers (Atkinson and Nevill, 1998). In this study, CVs were generally low in all comparisons. Even though there is no universally accepted scale for interpreting CV, in the health and medical area, a CV that is lower than 5% is generally deemed acceptable (Machin et al., 2007). CV in the squat was < 5% in 75% of all outcomes. Similar was observed for the deadlift, as CVs for 65% of all outcomes were < 5%. Furthermore, all CVs that were higher than 5% in the first comparison, decreased 0.7–2.9% in the second comparison. This finding is in line with that of Hopkins et al. (2001), who concluded that adding a practice session may decrease CV on average by 1.2% due to the learning effect. CVs tended to be higher at 90% of 1RM, compared with lower loads. However, it should be considered that the participants performed lifts at 90% of 1RM last in a given exercise, as the loads were progressed from lowest to highest in each session. Therefore, this might not be explained by the load *per se*; rather, it might be a consequence of fatigue accumulated earlier in the exercise tests.

SEM was also generally low for most outcomes. Interestingly, in the squat exercise, SEM for mean velocity (the most practically relevant outcome in VBT; Sánchez-Medina and González-Badillo, 2011), ranged 0.03–0.06 m/s when comparing the results of the first and second testing session. When comparing the results from the second and third sessions, SEM for this outcome was reduced to 0.03 m/s for all loads. SEM for mean velocity reported herein is well below the changes for this outcome reported following a training intervention. For example, increases in mean velocity following a training intervention exhibited against loads lifted slower or faster than 1.00 m/s, ranged 0.08–0.13 m/s (Galiano et al., 2020).

Thus far, two studies explored this topic, while using a design similar to the design used in the present study (Chéry and Ruf, 2019; Orange et al., 2020). Orange et al. (2020) included 29 youth rugby league players, who performed the squat and bench press exercises with loads 20–90% of 1RM on two different testing sessions. The authors found good-to-excellent reliability of velocity and power assessed by the GYM system at loads in the 40–90% of 1RM range. Reliability tended to be lower at 20% of 1RM. In the current study, we did not observe a tendency toward lower reliability for the lowest load. However, it needs to be considered that the lowest load used in our study was 30% of 1RM (compared to 20% of 1RM in the Orange et al. (2020) study). It is possible that reliability starts to decrease at loads below 30% of 1RM. Chéry and Ruf (2019) used the deadlift exercise and loads ranging 20–100% of 1RM for assessing velocity and power. This study reported that velocity and power assessed by the GYM system had good reliability at loads of 60, 80, and 90% of 1RM. At loads of 20, 40, and 100% of 1RM, velocity and power tended to be less reliable. Our results are in agreement with their findings regarding the reliability of velocity and power at loads from 60 to 80% of 1RM. However, we did not utilize loads of 20, 40, and 100% of 1RM, and therefore, we cannot make direct comparisons with the findings of Chéry and Ruf (2019) study in regard to these loads.

The main limitation of this study is that velocity and power in the squat and deadlift were assessed in the same session. This might have impacted the results given that the fatigue induced during the deadlift might have impacted participants’ performance in the squat. Indeed, in the post-study interviews, we asked the participants post-testing if they felt that their squat performance was affected by the deadlift performance, and some of them indicated that this might have been the case. We opted for such an approach to increase the practical applicability of the findings, given that individuals performing resistance training are likely to perform more than one exercise per session. Additionally, velocity and power were tested using a protocol where the load was progressively increased each set. Therefore, fatigue inducted during one set might have impacted participants’ performance in subsequent sets. We attempted to minimize potential confounding with this approach, by providing the participants with 10 min of rest between exercises and 3 min of rest between sets. Finally, the warm-up prior to testing was self-selected. Since our participants were experienced in resistance training, our approach was to allow them to prepare for testing sessions according to their usual habits. In light of their habits, some individuals might need more warm-up time dedicated to their lower body, while others might need to focus more on their upper-body musculature to achieve optimal exercise performance. These inter-individual differences in warm-up routines may have contributed to testing performance results. However, as related to our research question, the participants were instructed to keep their warm-up routine consistent across all sessions.

Based on the results of this study, it can be concluded that the reliability of velocity and power assessed by the GYM system is moderate-to-excellent for the deadlift exercise and good-to-excellent for the squat exercise. Reliability velocity and power generally did not seem to improve with a familiarization session.

## Data Availability Statement

The dataset generated for this study is available from the corresponding author upon a reasonable request.

## Ethics Statement

The study protocol was reviewed and approved by the Committee for Scientific Research and Ethics of the Faculty of Kinesiology at the University of Zagreb (approval number: 74/2020). The participants provided their written informed consent to participate in this study.

## Author Contributions

JG and PM conceived the idea and designed the study. BS collected the data. PM supervised the data collection. JG, ZP, and PM analyzed the data. JG interpreted the results and drafted the manuscript. PM, ZP, and BS contributed to interpreting the results and writing the manuscript. All authors reviewed and approved the draft manuscript and all its revisions.

## Conflict of Interest

The authors declare that the research was conducted in the absence of any commercial or financial relationships that could be construed as a potential conflict of interest.
